# Transcriptomic and metabolomic analysis of yellow leaf mutant variation in *Taxus cuspidata*

**DOI:** 10.3389/fpls.2025.1642681

**Published:** 2025-09-12

**Authors:** Shuo Zhang, Duo Liu, Lihui Zhang, Yanwen Zhang

**Affiliations:** ^1^ College of Life Sciences, Changchun Normal University, Changchun, China; ^2^ School of Agricultural, Liaodong University, Dandong, China

**Keywords:** *Taxus cuspidata*, metabolomics, transcriptomics, yellow leaf, green leaf

## Abstract

During the cultivation of *Taxus cuspidata*, significant differences in leaf coloration between different types have emerged, which is of great significance for variety breeding. This study employed transcriptomic and metabolomic analyzes to identify key genes and metabolites associated with leaf color variation between the yellow leaf type and the green leaf type. The results showed: (1) Metabolites such as kaempferol 3-p-coumaroylglucoside, quercetin-3’-glucuronide, kaempferol-3-O-rutinoside, Ridiculuflavone D, phaeophorbide b, and paclitaxel were significantly higher in the yellow leaf type compared to the green leaf type, while the content of tetrapyrrole compounds was significantly lower in the yellow leaf type.(2) Transcriptomic analysis indicated that genes involved in carotenoid synthesis, flavonoid synthesis, and chlorophyll degradation, such as F3H, FLS, ZEP, PSY, and FLN, were significantly upregulated in the yellow leaf type compared to the green leaf type. In contrast, genes involved in chlorophyll biosynthesis (GLK, SGR) and anthocyanin synthesis (DFR) were significantly downregulated. qRT-PCR analysis further validated these results. (3) Integrative transcriptomic and metabolomic analysis revealed significant positive correlations between F3H, FLS, FLN genes and flavonoid compounds, and between GLK, SGR genes and the reduction in tetrapyrrole compounds, promoting chlorophyll and chloroplast degradation. These findings suggest that the acquisition of yellow leaf traits in *Taxus cuspidata* is mainly achieved by enhancing upstream flavonoid biosynthesis pathways and downstream chlorophyll degradation pathways, including phenylpropanoid biosynthesis, flavonoid biosynthesis, and chlorophyll degradation, while limiting the downstream anthocyanin biosynthesis pathway and related processes.

## 1 Introduction

Non-green leaf color variants, often referred to as ornamental foliage plants, represent important aesthetic traits in landscaping, similar to flower coloration. Unlike the transient nature of flower color, stable variations in leaf color can impact photosynthetic efficiency and are generally considered maladaptive in natural environments (Fan et al., 2024). However, in horticultural breeding, variations in leaf coloration enhance the ornamental value of plants, making them more attractive for landscaping. In many countries, such as those in Europe and North America, ornamental foliage trees account for over 30% of the plantings in some urban green spaces (Özbayram and Çiçek, 2018).

Leaf color is primarily determined by the relative concentrations of chlorophylls, anthocyanins, and carotenoids (Shen et al., 2018). Stable variations in leaf color involve complex regulatory mechanisms of pigment synthesis and degradation, significantly influenced by environmental factors such as light intensity, temperature, hormones, and sugar metabolism (Dong and Lin, 2020). Historically, research has focused more on flower color variation, with relatively limited studies on the molecular mechanisms underlying leaf color variation, especially cases independent from flower pigmentation. Recent findings highlight the pivotal role of the MBW complex, composed of MYB, bHLH, and WD40 proteins, in regulating anthocyanin biosynthesis (Lloyd et al., 2017). For instance, Li et al. (2024) discovered that an R2R3-MYB transcription factor activated anthocyanin biosynthesis, leading to red leaf coloration in a poplar mutant. Additionally, Wang et al. Wang et al. (2023) used integrated transcriptomic and metabolomic analysis to reveal the major regulatory mechanisms of leaf color variation in Fraxinus excelsior. Other studies have shown that leaf color variation is primarily influenced by dynamic changes in photosynthetic pigments: chlorophyll degradation can cause leaves to transition from green to yellow, while the accumulation of carotenoids, such as lutein and β-carotene, is a key factor in the formation of yellow leaves (Hörtensteiner, 2009; Wang et al., 2022). Moreover, anthocyanin accumulation leads to red or purple leaf coloration, commonly seen in autumn foliage plants (Gould, 2012). However, most research has centered around anthocyanin-related changes, while the molecular mechanisms of yellow leaf formation remain largely unexplored.

With the advancement of high-throughput sequencing technologies, integrated transcriptomics (RNA-Seq) and metabolomics analyzes have become powerful tools for studying leaf color variations (Chen et al., 2024). By analyzing the expression patterns of key genes in pigment biosynthesis pathways and the dynamic changes of secondary metabolites, the molecular regulatory networks governing different leaf color types can be systematically elucidated. In the study of leaf color variation, yellow leaf traits may involve the upregulation of chlorophyll degradation pathways, enhancement of carotenoid biosynthesis, and changes in cellular structure. Flavonoids, including flavonols and other derivatives, have been implicated in promoting leaf yellowing across various plant species (Martens et al., 2010; Zhao et al., 2012; Zhao and Tao, 2015). For example, the accumulation of flavonoids and flavonols in ginkgo (Ginkgo biloba) and Macadamia leaves (HAES344) was associated with the yellowing phenomenon (Shi et al; Yang et al., 2024). Nevertheless, the specific molecular mechanisms underlying these changes require further investigation.

*Taxus cuspidata* (Japanese yew) is a rare and endangered species native to northeastern China, Japan, and Korea, with high medicinal value due to its high content of paclitaxel (Zhang, 2021). Thanks to its outstanding cold resistance and evergreen foliage, it has been widely used in East Asian landscapes, particularly prized for its red berries produced in the autumn. However, the traditional wild types exhibit uniform green foliage, limiting their ornamental diversity. Recently, through mutagenesis screening and artificial hybridization, several new leaf color variants have been developed, including golden yellow, orange-red, dark green, and types that transition from yellow to red in autumn (Wang et al., 2019). Among these, yellow leaf variants have garnered significant attention for their unique visual appeal.

Leaf color variation in gymnosperms, particularly the formation of yellow-leaf phenotypes, is governed by complex genetic networks involving chlorophyll degradation, carotenoid accumulation, and other physiological processes. Studies on certain gymnosperms have shown that the formation of yellow leaves is closely related to the balance between carotenoid synthesis and chlorophyll degradation. For instance, Li et al. (2018) found that the upregulation of genes such as F3H (flavanone 3-hydroxylase) and FLS (flavonol synthase) in yellow leaf types promotes carotenoid biosynthesis, while GLK (Golden-like), a gene involved in chlorophyll degradation, is downregulated. Similarly, Wu et al. (2020b) observed that the expression changes of chlorophyll degradation-related genes are closely associated with yellow leaf formation, further supporting the role of these genes in regulating leaf color (Li et al., 2018; Wu et al., 2020b).

While the molecular mechanisms underlying yellow leaf formation in gymnosperms have been studied to some extent, the genetic regulation of yellow leaf phenotypes in Taxus cuspidata remains unexplored. In our previous studies, We observed significant changes in both carotenoid and chlorophyll content in the yellow leaf phenotype compared to the green leaf type. These physiological differences suggested that the balance between carotenoids and chlorophyll might play an important role in the formation of the yellow leaf phenotype. This result provides a foundation for our subsequent research.

In Taxus cuspidata, leaf color variation occurs as a transition from green to yellow, with different shades including bright yellow, golden yellow, and orange. These variations are primarily attributed to differences in carotenoid content within leaf cells. The variations in leaf color intensity can be treated as distinct ornamental cultivars for horticultural purposes. In this study, we focused on a golden-yellow variant, known as the “Danjin” cultivar, which was identified through our research. This cultivar is characterized by its strong adaptability and widespread cultivation, particularly in northeastern China, making it an ideal subject for exploring the molecular mechanisms of leaf color formation. By investigating this cultivar, we aim to provide both fundamental insights into pigment regulation and practical guidance for the breeding and cultivation of ornamental Taxus cultivars.

Nevertheless, the molecular basis underlying leaf color variation in *Taxus cuspidata* remains poorly understood, with little systematic analysis of pigment metabolism pathways and regulatory networks, hindering the breeding and application of new cultivars. In this study, we utilized Illumina HiSeq-based transcriptomic analysis and combined it with metabolomic profiling to investigate the regulatory mechanisms of leaf color formation between yellow leaf and green leaf types of Taxus cuspidata. By identifying key genes involved in pigment metabolism pathways and validating their expression patterns through qRT-PCR, alongside metabolite accumulation analysis, we aimed to unravel the core regulatory networks responsible for leaf color variation.

## 2 Materials and methods

### 2.1 Plant materials

**Figure 1:**
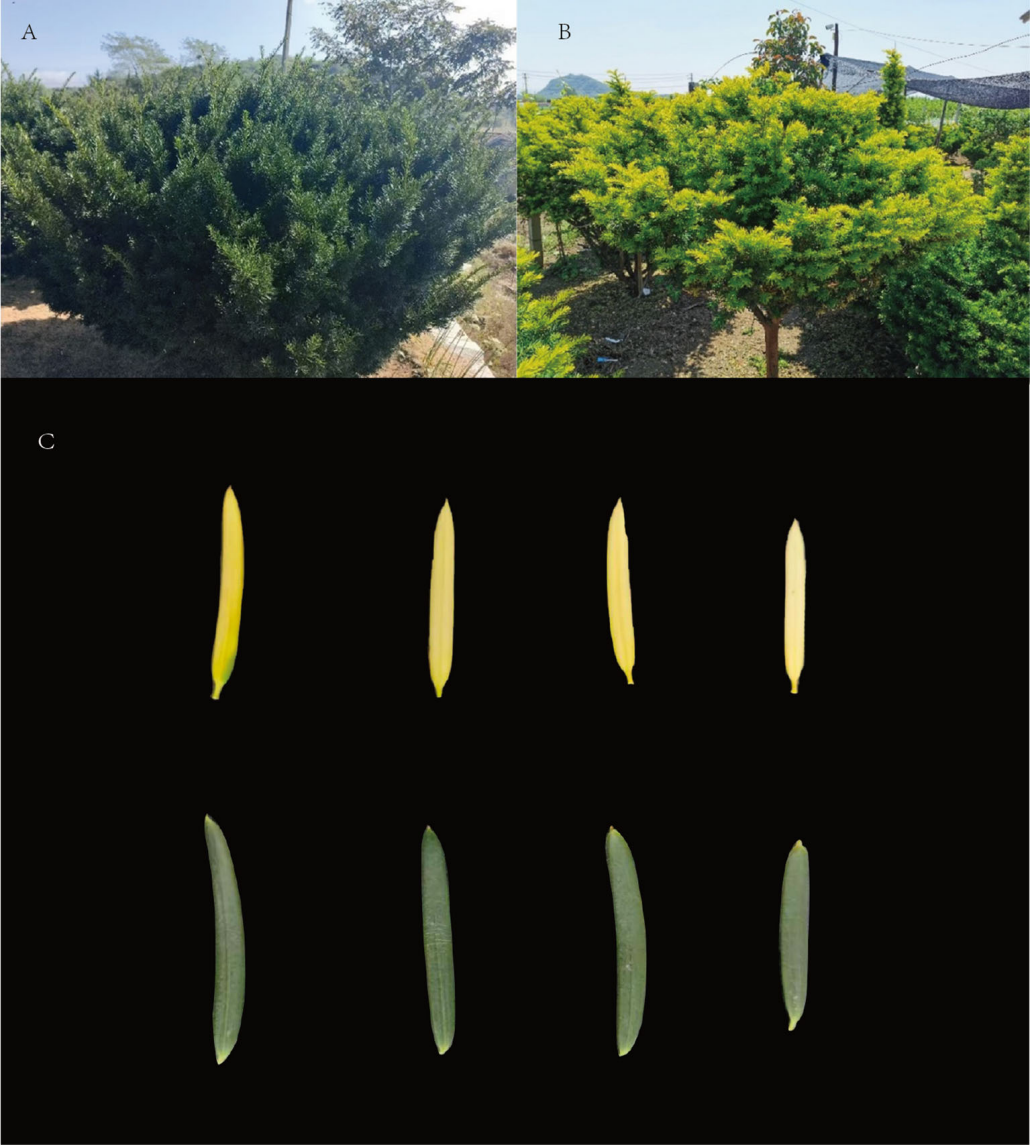
**(A)** Green-leaf plant **(B)** Yellow-leaf plant **(C)** Comparison of leaf-color phenotypes.

Experimental materials were collected from a *Taxus cuspidata* plantation located at the Dandong Jingou Science and Technology Ecological Park (E123°62′, N40°08′), Liaoning Province, China. We selected green leaf types from healthy, similarly sized trees growing under uniform environmental conditions, along with yellow leaf types identified from variant individuals (**Figure 1**). All sampled leaves were fully expanded, current‐year leaves that had attained stable coloration by the time of collection. After collection, leaves were rinsed with purified water, immediately frozen in liquid nitrogen, and stored at -80°C until RNA extraction.

### 2.2 Experimental methods

#### 2.2.1 RNA extraction, library construction, and sequencing

Total RNA was extracted from leaf samples using a standard protocol and its integrity and concentration were verified using an Agilent 2100 Bioanalyzer (Agilent Technologies, CA, USA).mRNA was enriched using Oligo(dT) magnetic beads and fragmented to appropriate sizes, followed by cDNA synthesis with random hexamer primers. Subsequent steps included end repair, A-tailing, adaptor ligation, size selection, PCR amplification, and purification to prepare the final library. Library quantification was performed using a Qubit fluorometer and real-time PCR. Qualified libraries were pooled and sequenced using the Illumina NovaSeq platform.

#### 2.2.2 Data quality control, sequence alignment, and expression quantification

Raw reads were processed using fastp to generate clean reads by removing adapters, low-quality sequences, and reads containing poly-N. Quality metrics such as Q20, Q30, and GC content were calculated. Clean reads were aligned to the *Taxus chinensis* reference genome (NCBI database) using HISAT2 v2.0.5. For transcriptomic analysis, reads were mapped to the *Taxus chinensis* reference genome due to the unavailability of a complete *Taxus cuspidata* genome at the time of analysis. Although *Taxus chinensis* and *Taxus cuspidata* are closely related species, we acknowledge that this may introduce some biases, particularly in the identification of *Taxus cuspidata*-specific genes. In future studies, the availability of a *Taxus cuspidata* genome assembly would improve the accuracy of gene annotations and reduce potential species-specific biases in gene expression analysis. Unigenes were annotated based on comparisons with the NR, Swiss-Prot, COG, KOG, egg NOG, and KEGG databases. Feature Counts (v1.5.0-p3) was used to count reads mapped to each gene, and gene expression levels were calculated as FPKM values.

#### 2.2.3 Differential expression and functional enrichment analyzes

Differential expression analysis between yellow leaf and green leaf types was performed using the DESeq2 R package (v1.20.0). The Benjamini-Hochberg method was applied for multiple test correction, setting thresholds of Padj ≤ 0.05 and |log2FoldChange| ≥ 1 to identify significantly differentially expressed genes (DEGs).

GO enrichment analysis of DEGs was conducted using clusterProfiler (v3.8.1), with KEGG pathway enrichment analysis performed simultaneously to identify significantly enriched metabolic and signaling pathways.

The transcriptome sequencing data from this study will be made publicly available in the NCBI SRA database upon formal publication of the paper. Data will be made available for peer-reviewed use upon reasonable request.

#### 2.2.4 Metabolite extraction and detection

Leaf tissue (100 mg) was ground under liquid nitrogen and extracted with 500 μL of 80%methanol-water solution. Samples were vortexed, incubated on ice, and centrifuged at 15,000 g for 20 min at 4°C. Supernatants were collected, diluted to a final methanol concentration of 53%, and analyzed by liquid chromatography-mass spectrometry (LC-MS). Equal volumes of each sample were pooled to create quality control (QC) samples.

PCA and PLS-DA analyzes were performed using meta X software to evaluate data quality and discriminate between groups. Metabolites with VIP > 1, P < 0.05, and fold change (FC) ≥ 2 or ≤ 0.5 were considered significantly differentially accumulated.

#### 2.2.5 qRT-PCR validation

Specific primers were designed based on candidate genes identified from the transcriptome data using Primer Premier 5.0 (Primer sequences are listed in **Table 1**). cDNA was synthesized from extracted RNA using a reverse transcription kit. qRT-PCR was performed using SYBR Premix Ex Taq in a 20 μL reaction volume. Cycling conditions were: 95°C for 30 s, followed by 40 cycles of 95°C for 5 s and 60°C for 30 s. Melt curve analysis confirmed primer specificity. Relative gene expression was calculated using the 2^−ΔΔCt method, with 18S rRNA as the internal reference.

**Table 1:** Sequences of primers used in qRT-PCR.

Primer	Forward primer (5’ to 3’)	Reverse primer (5’ to 3’)
PSY	TGTCAAGCCTGAAATTGCCTCTCC	CTCAGCACAGACTTCGCCACAG
ZEP	GTCCTAATGGCGTCTCTGCTCTTG	CCTTTGGCGGCATGTTTGTTTCTC
FLN1	AACGAAGACAGGCAGGCGAAAC	TTCTCTCCCTTTGCTGTTGCTTCC
F3H	ACGTCCCCAAATGTCCAAGGTTTC	AGCCATCCTGTTCACAAGCACTTC
GLK1	CCTTGCTGCTCGCTGAAGTCTC	TGGACGCTATCTTTACGCATTCGG
SGR	TGGGAGTGGATGGAGAGAAGCAAG	ACTGTGCCTTGTTGATGGTTTGGG

#### 2.2.6 Measurement of total flavonoids, carotenoids, and chlorophyll

The total flavonoid content was determined using the NY/T 1295-2007 method (Determination of Total Flavonoid Content in Buckwheat and Its Products). A sample of dried leaves was weighed and extracted with 80% methanol solution at 65°C for 30 minutes. The extract was then filtered to obtain the supernatant. To the extract, 0.5 mL of aluminum chloride (AlCl_3_) solution was added, followed by 4.5 mL of methanol. After incubating at room temperature for 30 minutes, the yellow-colored complex formed was measured by spectrophotometry at 420 nm. The flavonoid content was quantified by comparing the absorbance with a quercetin standard curve. Chlorophyll content was determined using the acetone-ethanol colorimetric method. Leaf samples were ground and mixed with a 90% acetone solution and incubated at 4°C overnight. The chlorophyll extract was then filtered, and absorbance was measured at 645 nm and 663 nm. The total chlorophyll content was calculated based on the absorption coefficients of chlorophyll a and chlorophyll b. Carotenoid content was determined using the acetone-ethanol colorimetric method as well. Leaf samples were ground and mixed with 80% acetone solution. After incubation at 4°C for 30 minutes, the mixture was filtered and the absorbance was measured at 450 nm. The carotenoid content was calculated using a standard equation based on the absorbance.

These methods allow for the accurate determination of flavonoids, chlorophylls, and carotenoids, providing a comprehensive understanding of the pigment composition and their roles in leaf coloration.

#### 2.2.7 Statistical analysis

Transcriptomic and metabolomic analyzes were conducted with four biological replicates. Metabolomics data were processed using meta X for PCA, PLS-DA, and statistical testing. Correlation analysis, clustering heatmaps, volcano plots, and pathway enrichment analyzing were performed using R software. Gene expression levels were calculated using HTSeq and DESeq2. Omics correlation analyzes were performed using mixOmics, and visualized using Cytoscape to explore gene-metabolite interaction networks. Gene expression differences were analyzed using qRT-PCR and t-tests, with statistical significance indicated by p < 0.05 and p < 0.01, where asterisks represent significance (p < 0.05) and extreme significance (p < 0.01). Results were visualized in bar graphs.

## 3 Results

### 3.1 Transcriptomic analysis of leaf color variation in *Taxus cuspidata*

#### 3.1.1 Quality of transcriptome sequencing and gene annotation

To investigate the molecular mechanisms underlying leaf color variation in *Taxus cuspidata*, we performed transcriptome sequencing and assembly for eight samples representing green leaf and yellow leaf types. In total, 309,696,458 clean reads were obtained, with sequencing error rates below 0.01%. The average Q20 and Q30 values were 98.48% and 95.51%, respectively, and the average GC content was approximately 44%. Clean reads were mapped to the *Taxus chinensis* reference genome with mapping rates ranging from 78.78% to 79.39%, indicating high-quality sequencing data suitable for downstream analyzes. Sequencing quality metrics are summarized in **Table 2**.

**Table 2:** Overview of sequencing quality metrics.

Sample	Raw_bases	Clean_bases	Error_rate	Q20	Q30	GC_pct
Y_1	6.46G	6.35G	0.01	98.52	95.58	44.02
Y_2	6.35G	6.19G	0.01	98.34	95.06	44.41
Y_3	7.20G	6.85G	0.01	98.58	95.79	44.47
Y_4	6.90G	6.75G	0.01	98.37	95.12	44.17
G_1	6.79G	6.65G	0.01	98.55	95.74	44.76
G_2	6.96G	6.85G	0.01	98.43	95.37	44.71
G_3	6.67G	6.52G	0.01	98.51	95.56	44.70
G_4	6.94G	6.82G	0.01	98.61	95.88	44.82

#### 3.1.2 Gene differential expression analysis of transcriptomic data

**Figure 2:**
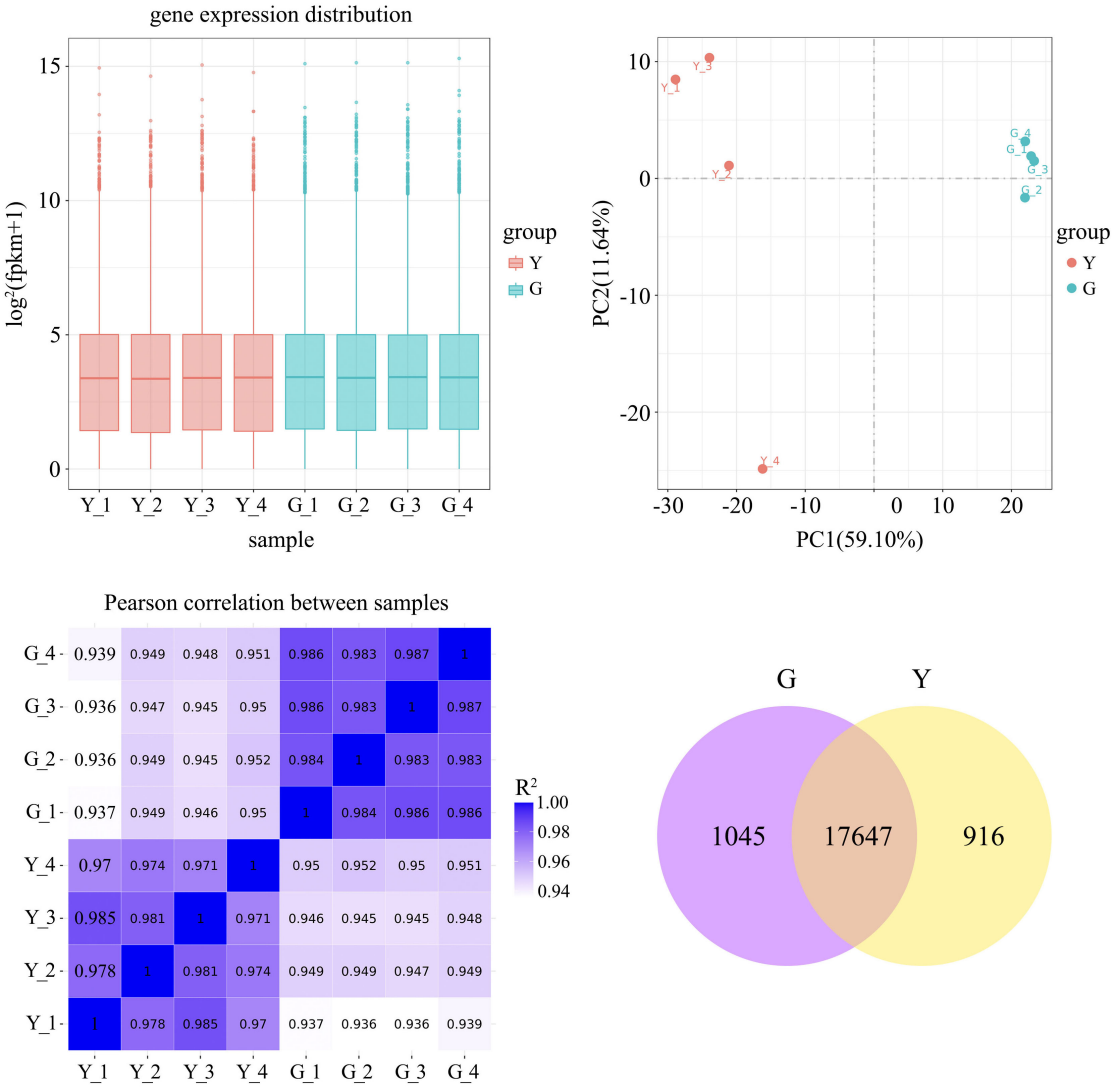
**(A)** Box-plot of gene-expression distribution for each sample (X-axis: sample name; Y-axis: log_2_ (FPKM + 1)) **(B)** Principal-component analysis (PCA) plot (X-axis: PC1; Y-axis: PC2) **(C)** Pearson correlation matrix among samples (Y = yellow-leaf; G = green-leaf) **(D)** Numbers of specifically expressed and co-expressed genes between green-leaf and yellow-leaf *Taxus cuspidate*.

To ensure the accuracy of the subsequent analysis, we first corrected the sequencing depth and then the length of the gene or transcript to obtain the FPKM value of the gene, and the expression distribution was calculated based on the FPKM value. From the distribution, it can be seen that the gene expression is more homogeneous among different samples, which means that these results can be used for subsequent differential gene analysis (**Figure 2A**). Principal component analysis (PCA) and pearson correlation coefficient analysis clustered the samples based on expression levels. By PCA analysis between these samples, the results showed that the four biological replicates of the same sample were distributed in the same quadrant (**Figure 2B**). Pearson correlation coefficient analysis showed that the correlation of every two samples was above 0.85 (**Figure 2C**), which indicated that the samples were well replicated. Comparing the cDNA libraries of normal northeastern red bean sugi green leaves and yellow leaves, 18692 and 18563 genes were identified, respectively, and a total of 17647 genes were co-expressed in the normal green leaves and variant yellow leaves of northeastern red bean sugi, accounting for 89.9% of the total number of genes. Among them, 1045 genes were specifically expressed in the green leaves of NE red bean trees, accounting for 5% of the total, while 916 genes were specifically expressed in the yellow leaves of NE red bean trees, accounting for 4.6% of the total (**Figure 2D**).

#### 3.1.3 Identification of differentially expressed transcription factors

Among the differentially expressed genes (DEGs) between yellow and green leaf types, 692 transcription factors (TFs) were identified, mainly belonging to the bHLH, MYB, WRKY, NAC, ERF, and GATA families. Of these, 588 TFs exhibited significant expression differences (|log2FoldChange| ≥ 1, padj < 0.01). Notably, bHLH family members (KI387_035757 and KI387_019382) showed the highest upregulation (log2FoldChange > 12). Several NAC and ERF family members, as well as one GATA family member (KI387_005364), also exhibited significant expression changes.

#### 3.1.4 Functional enrichment analysis of DEGs

**Figure 3:**
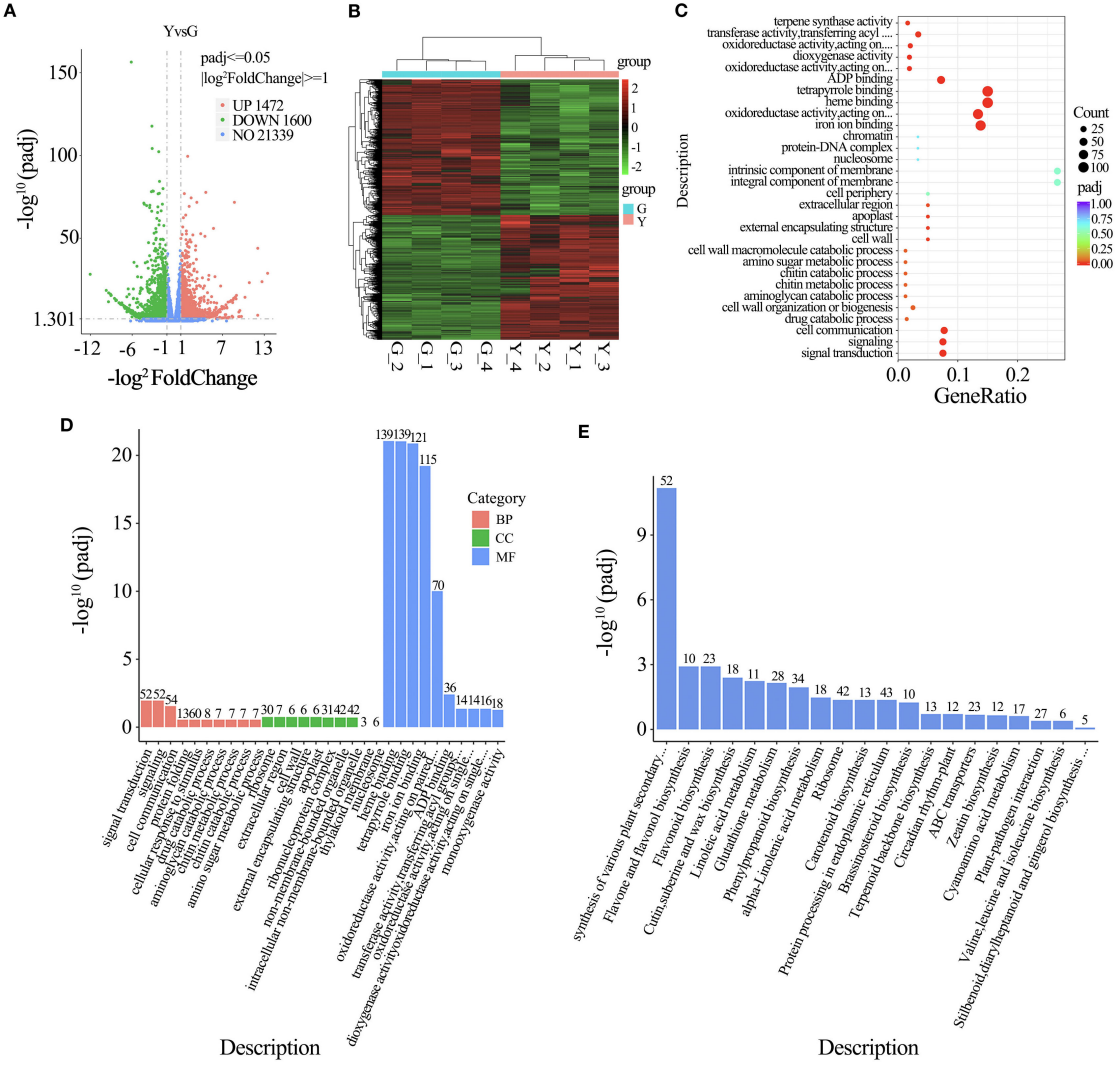
**(A)** Volcano plot of differentially expressed genes in *Taxus cuspidata* (X-axis: log_2_ Fold Change; Y-axis: –log_10_ adj-*p*). Dashed lines denote the threshold used for DEG selection. **(B)** Cluster heat-map of DEGs (X-axis: sample name; Y-axis: FPKM values of DEGs after normalization). **(C)** KEGG enrichment dot plot (X-axis: proportion of DEGs annotated to each pathway; Y-axis: KEGG pathway). Dot size indicates the number of DEGs mapped to the pathway; color from red to purple reflects enrichment significance. **(D)** GO enrichment bar plot (X-axis: GO term; Y-axis: –log_10_ adj-*p*). Colors distinguish different functional categories. **(E)** KEGG enrichment bar plot (X-axis: KEGG pathway; Y-axis: enrichment significance expressed as –log_10_ adj-*p*).

The differentially expressed genes in yellow leaf and green leaf were screened using DESeq, and the screening threshold was set to Padj<0.05. There were 3072 differentially expressed genes in yellow leaf and green leaf, of which 1472 were up-regulated genes and 1600 were down-regulated genes (**Figure 3A**). The differential genes of all comparison groups were taken and set as the differential gene set, and the differential gene set was analyzed by clustering to cluster the genes with similar expression patterns. The mainstream hierarchical clustering was used to cluster analyze the FPKM values of the genes, and the rows (rows) were homogenized (Z-score), which demonstrated the differential expression patterns of the gene sets of two different samples of Northeast red bean curd leaves, and investigated the pattern of the distribution of the differential gene expression (**Figure 3B**).

In order to elucidate the role of differentially expressed genes in the color change of NE red bean tree leaves, GO annotation and functional classification were performed on all differential gene sets. According to the results of GO enrichment analysis, the differentially expressed genes of YELLOW leaf and GREEN leaf were mainly focused on signal transduction, cell signaling molecules, cell communication, and protein folding in biological processes. In cellular components, GO terms were mainly concentrated in ribosomes, ribosomal proteins and so on. In molecular functions, the differentially expressed genes were significantly enriched in heme binding, photosynthetic response, tetrapyrrole binding, ironion binding, and oxidoreductase activity acting on paired (**Figure 3D**).

Then, KEGG pathway enrichment analysis was performed using the differentially expressed genes to analyze the key metabolic pathways enriched in the leaf color changes of NEH. Based on the results of KEGG metabolic pathway enrichment analysis, we found that the differentially expressed genes of green leaf and yellow leaf were significantly enriched in the metabolic pathways of flavonoid biosynthesis, flavonoid and flavonol biosynthesis, phenylpropanoid biosynthesis, glutathione metabolism, and carotenoid biosynthesis. Functional enrichment analysis indicated that the differentially expressed genes played important roles in the color changes of NEH leaves (**Figures 3C, E**).

### 3.2 Metabolomic analysis of leaf color variation in *Taxus cuspidata*

**Figure 4:**
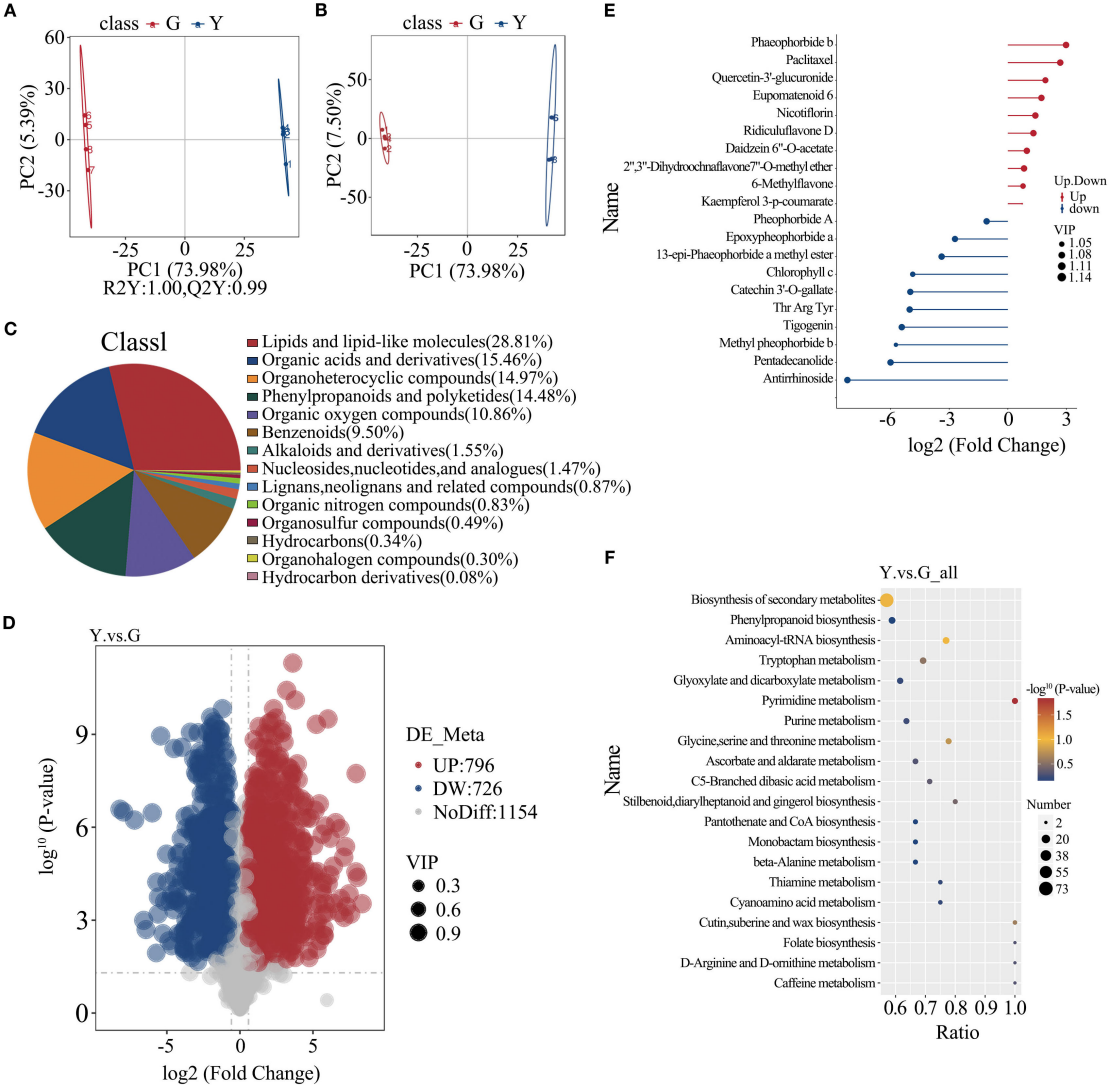
**(A)** Principal-component analysis (PCA) score plot **(B)** PLS-DA score plot with permutation validation **(C)** Pie chart of metabolite classes **(D)** Volcano plot of differential metabolites **(E)** Lollipop plot of differential metabolites **(F)** KEGG enrichment bubble plot.

Based on the results of principal component analysis (PCA), yellow leaf type and green leaf type were shown as two groups (**Figure 4A**). The PLS-DA model in this study involved pairwise comparisons of differential metabolite contents to assess the differences between yellow leaf and green leaf (R2Y=1,Q2Y=0.99; **Figure 4B**) indicating that this model is stable and reliable for further screening of metabolite differences. After quality validation, a total of 1522 metabolites were identified. The results showed that 796 metabolites were significantly up-regulated and 726 were significantly down-regulated in yellow leaf compared to green leaf. Next, the chemical classification of the total metabolites identified in yellow leaf and green leaf was counted, and it was found that phenylpropane and polyketide substances, precursors for flavonoid synthesis, occupied 14.48% (**Figures 4C, D**). Through the differential metabolite matchstick diagram, we clearly found that yellow leaf showed a significant up-regulation of various flavonoids and isoflavonoids metabolites, such as kaempferol 3-p-coumarate, quercetin-3’-glucuronide, kaempferol-3-O-rutinoside, and Ridiculuflavone D in comparison to green leaf, and the demagnetized chlorophyll-like compounds Phaeophorbide b also showed up-regulation, while metabolites such as Chlorophyll c and Pheophorbide A showed down-regulation (**Figure 4E**). Interestingly we found the anticancer drug paclitaxel among the significantly up-regulated metabolites, with the content of paclitaxel in yellow leaf reaching about 7 times that of green leaf compared to green leaf. These metabolites were enriched in 46 pathways, including secondary metabolite biosynthesis, flavonoid biosynthesis, flavonoid, and flavonol biosynthesis (**Figure 4F**). Taken together, we found that the accumulation of flavonoids, with fewer tetrapyrrole compounds associated with chlorophyll, was significantly active in the metabolomics analysis, which provided an important basis for subsequent analyzes.

**Figure 5:**
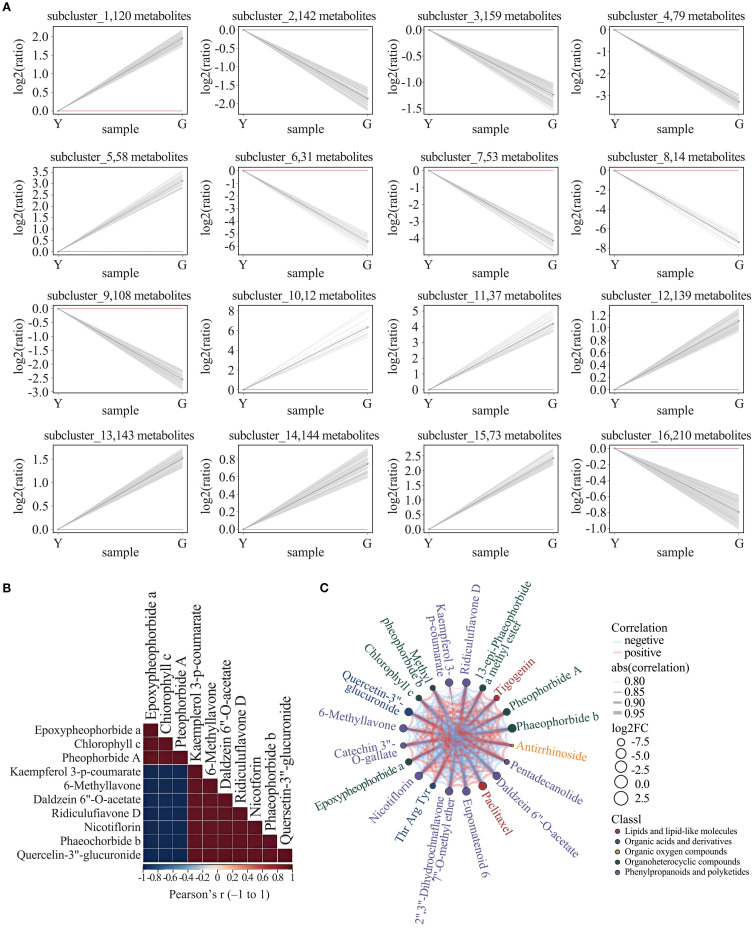
**(A)** K-means clustering of differential metabolites. Gray lines = individual metabolites; Blue line = subcluster mean. **(B)** Correlation heat-map of differential metabolites. **(C)** Chord diagram illustrating relationships among differential metabolites.

To determine the abundance pattern of differential metabolites, we further performed Kmeans analysis and identified 16 clusters. Notably, there was an increasing trend of metabolites in Cluster 3, Cluster 16, based on the level of flavonoid content (**Figure 5A**). Cluster 3 consisted of 159 compounds, including flavonoids, organic acids, phenolic acids, and proteins. Cluster 16 consisted of 210 compounds, including flavonoids, alkaloids, phenolic compounds, amino acids and their derivatives, and some vitamins. In order to understand the synergistic or mutually exclusive roles of each metabolite in yellow leaf gold leaf traits, we performed differential metabolite correlation analysis on some metabolites, and the results showed that flavonoids and tetrapyrroles were negatively correlated during the formation of yellow leaf gold leaf traits, i.e., the accumulation of flavonoids and the degradation of tetrapyrroles jointly regulated the formation of yellow leaf traits. The correlation between different differential metabolites was visualized in the differential metabolite chord diagram. Amino acids can be found to synergistically regulate some chlorophyll-related metabolites, whereas a negative correlation with flavonoids is shown. Focusing the perspective to chlorophyll metabolites versus flavonoid metabolites it was found that chlorophyll degradation was synergistically regulated with flavonoid synthesis, showing a significant positive correlation. Based on the previous findings, we focused on the presentation of synergism of paclitaxel in YELLOW LEAF. Paclitaxel was found to be positively correlated with the high expression of flavonoids and the up-regulation of some chlorophylls, and negatively correlated with the degradation of some amino acids and organic oxides. The correlation matrix and the chord network are shown in **Figures 5B, C**.

### 3.3 Integrated transcriptomic and metabolomic analysis

**Figure 6:**
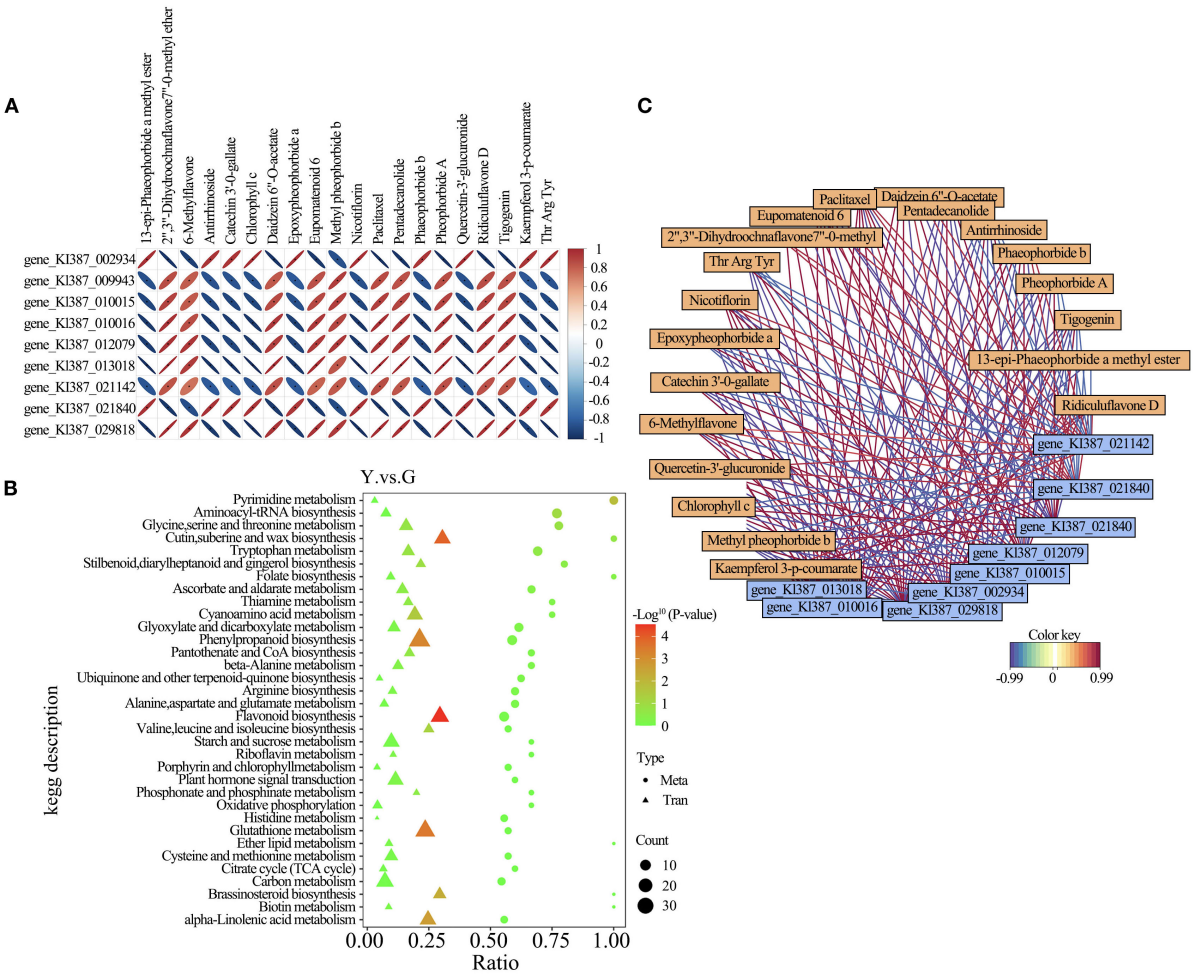
**(A)** Heat-map of correlations between differential metabolites and differentially expressed genes **(B)** KEGG enrichment bubble plot for differentially expressed genes and differential metabolites **(C)** Correlation network diagram.

In order to further explore the molecular response mechanism of leaf color in different types of NE Picea abies, we conducted a joint analysis of transcriptomic and metabolomic data. We screened the TOP10 differential genes and TOP20 differential metabolites for association analysis, and found that some genes related to chlorophyll content, such as GLK and SGR, synergize with tetrapyrrole compounds related to the regulation of the change of chlorophyll content as well as the synthetic degradation of chloroplasts. Whereas flavonoids, flavonoids as well as isoflavonoid compounds were highly associated with the C3H family, the differential gene F3H showed a positive correlation with most of the flavonoids, and the gene FLS showed the same synergistic trend. In contrast, DFR, which controls anthocyanin synthesis, showed positive correlation with tetrapyrrole compounds and negative correlation with flavonoids. The gene FLN, which promotes phenotypic yellowing, showed a negative correlation with tetrapyrrole compounds and a positive correlation with flavonoids flavonoids. (**Figures 6A, C**), we mapped all the obtained differential genes and differential metabolites to the KEGG pathway database separately to obtain their common pathway information, and identified the major biochemical pathways and signaling pathways in which the differential metabolites and differential genes were jointly involved, and found that they were mainly focused on the biosynthesis of flavonoids, riboflavin, and the biosynthesis of amino acid phenylpropanes, glutathione metabolism, pyrimidine metabolism, and the generation of some keratin waxes (**Figure 6B**).

### 3.4 Screening and validation of related differential genes

**Figure 7:**
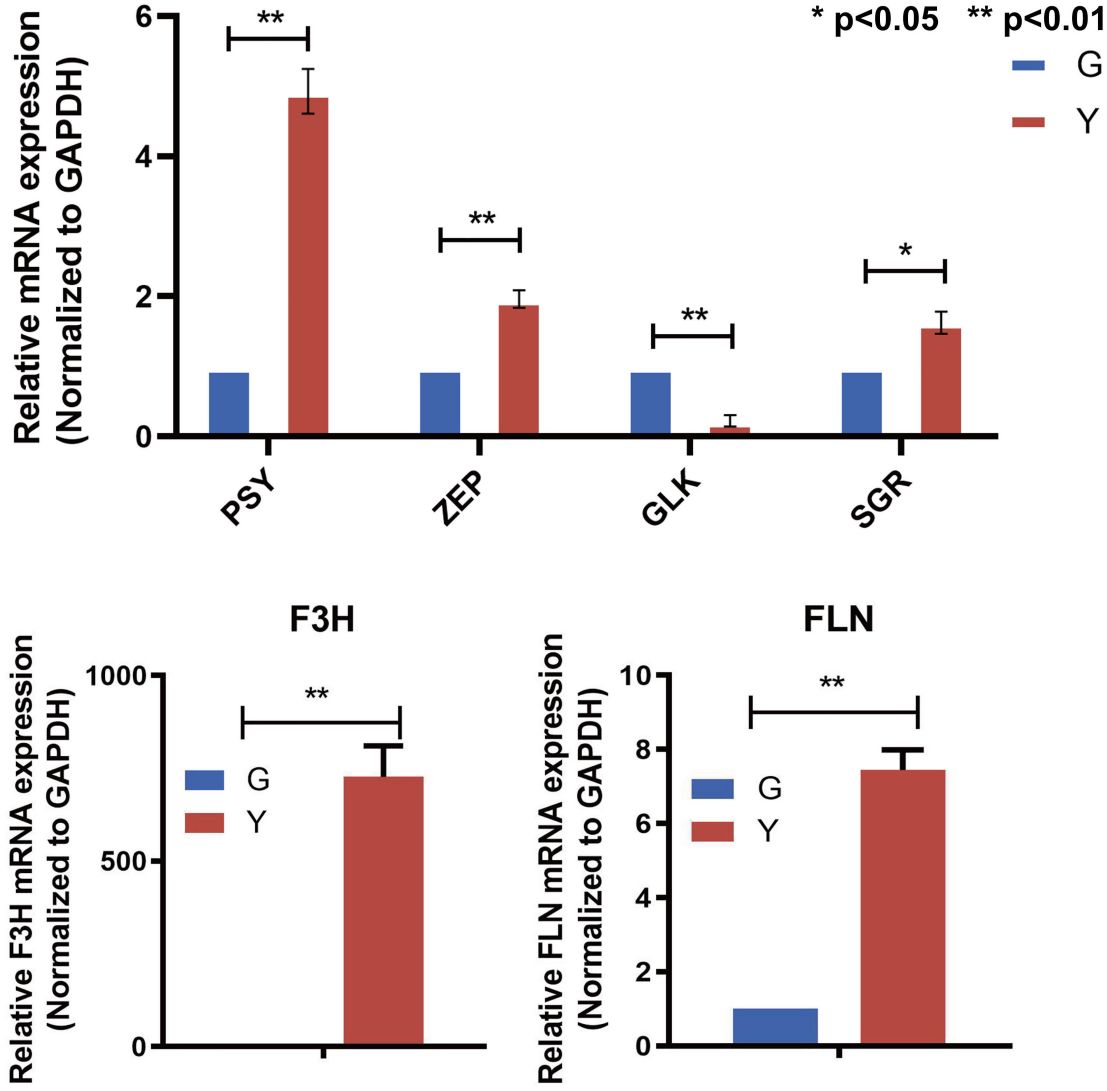
qRT-PCR validation of candidate genes. Top panel: PSY, ZEP, GLK and SGR (X-axis: Gene). Bottom panels: F3H and FLN (X-axis: Group, G and Y). Y-axis: Relative mRNA expression (2^-ΔΔCt, normalized to GAPDH). Bars show mean ± SD; significance is indicated as * p < 0.05 and ** p < 0.01 (two-tailed t-test).

Based on the results of the transcriptomics and metabolomics data analysis described above, we found that the differential genes or substances were mainly concentrated in flavonoids, carotenoids, and other related synthetic pathways. Therefore, we analyzed the genesrelated to flavonoids, carotenoids and others. By expression difference analysis, we screened three genes with high expression in yellow leaf (**Figure 7A**), SGR (gene_ KI387_012079), PSY (gene_KI387_013018), ZEP (gene_KI387_010016). and one gene with low expression in yellow leaf, GLK (gene_KI387_ 002934), were associated with chlorophyll degradation, carotenoid synthesis, and chlorophyll synthesis, respectively. The significant differential expression of these genes in the two leaf color types may be closely related to changes in leaf color.

The coloration of plant leaves is not only determined by two pigments, chlorophyll and carotenoids, alone, but some genes of other pathways also play a role in plant cells toregulate the formation of plant leaf color. We further analyzed the transcriptomic data for other genes related to the pathways that may regulate leaf color formation and found that two Unigenes were significantly up-regulated in yellow leaf (**Figures 7B, C**), one of which was flavanone 3-hydroxylase (F3H, flavonoid-3-hydroxylase) and the other gene was filamin (FLN, filamin). These two genes showed significant expression differences in different samples, suggesting that they may play an important role in leaf color changes.

### 3.5 Pigment content and proportion in yellow and green leaf phenotypes of *Taxus cuspidata*

**Figure 8:**
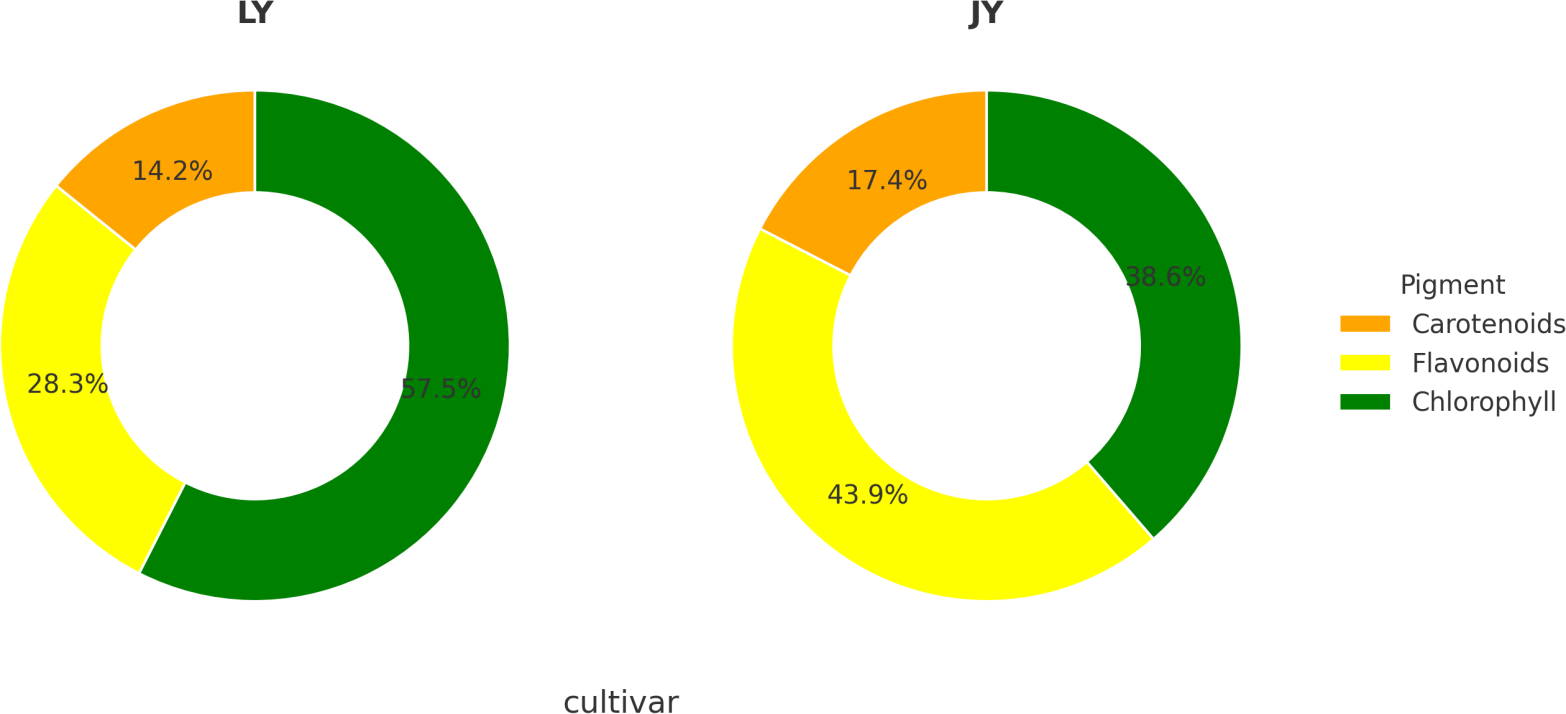
Proportions of leaf pigments in different leaf-color types of *Taxus cuspidata*.

Based on the results of multi-omics research, we designed measurements to determine the content of key pigments, including carotenoids, chlorophyll, and flavonoids, in the yellow-leaf and green-leaf phenotypes of *Taxus cuspidata*. The results showed that in the green-leaf variant, the total flavonoid content was 0.647%(Flavonoids (as rutin equivalents, fresh weight basis), with carotenoid and chlorophyll contents of 0.325 mg/g and 1.316mg/g, respectively. The total pigment content (sum of flavonoids, carotenoids, and chlorophyll) in the green-leaf phenotype was 2.288 mg/g. Flavonoids accounted for approximately 28.3% of the total pigment content. In the yellow-leaf variant, the total flavonoid content was lower at 0.223%(Flavonoids (as rutin equivalents, fresh weight basis), with carotenoid and chlorophyll contents of 0.089 mg/g and 0.20 mg/g, respectively. The total pigment content in the yellow-leaf phenotype was 0.512 mg/g. Despite the lower total flavonoid content in the yellow-leaf variant compared to the green-leaf, the proportion of flavonoids in the total pigment content increased to 43.9%. The relative proportions of pigments are shown in **Figure 8**.

## 4 Discussion

The molecular mechanisms underlying leaf color variation are primarily governed by the biosynthesis and degradation of pigments, chloroplast development, and the expression regulation of related genes (Chen et al., 2024). Key regulatory genes involved in the metabolic pathways of pigments such as chlorophylls, carotenoids, and flavonoids play central roles in determining leaf color (Huo et al., 2024). Environmental factors like light intensity and temperature further modulate gene expression, affecting pigmentation dynamics (Dong et al., 2019). Recent advances in transcriptomics and metabolomics have provided new insights into the molecular basis of leaf coloration. Many key genes have been confirmed to regulate pigment biosynthesis (Fang et al., 2019; Lu et al., 2019). The formation of yellow leaf traits is often associated with disruptions in photosynthetic machinery, altered chloroplast development, and the dynamic metabolism of chlorophylls and carotenoids (Wu et al., 2018). In *Taxus cuspidata*, our study identifies critical regulatory factors influencing the formation of the yellow leaf phenotype through combined transcriptomic and metabolomic analyzes.

### 4.1 Transcriptomic insights into yellow leaf formation

Genes involved in the flavonoid biosynthetic pathway are categorized into structural genes encoding biosynthetic enzymes and regulatory genes controlling structural gene expression (Gesell et al., 2015). In our analysis, 23 genes associated with the flavonoid biosynthetic pathway exhibited differential expression. Notably, F3H (flavanone 3-hydroxylase) was upregulated in the yellow leaf type, while other flavonoid-related genes like ROMT-15, CCOAOMT3, and DFR were downregulated. The transcriptomic data from this study were further supported by metabolomics analysis, which revealed that flavonoids, such as quercetin and kaempferol, were significantly accumulated in the yellow leaf type. This accumulation is likely facilitated by the upregulation of key genes such as F3H (flavanone 3-hydroxylase) and FLS (flavonol synthase). These findings highlight the critical role of flavonoid metabolism in regulating yellow leaf formation. Furthermore, the correlation between the upregulation of F3H and the increased levels of flavonols suggests a synergistic effect between gene expression and metabolite accumulation in driving the yellow coloration. qRT-PCR validation confirmed significantly higher expression of F3H in yellow leaves compared to green leaves, suggesting that overaccumulation of flavonols via F3H activation plays a critical role in yellow leaf formation.

Transcriptomics data also confirmed the key role of chlorophyll biodegradation genes in the formation of leaf color (Zhao et al., 2020; Liu et al., 2023). As the predominant pigment species in plants, the degradation as well as the synthesis pathways of chlorophyll have been extensively studied involving several functionally well-defined genes, including DVR, CHL, POR, HCAR, and hem among others (Cutolo et al., 2023). The transcriptomic data in this study showed that the expression levels of GLK and SGR were consistent with the decrease in chlorophyll content in yellow leaf. In the transcriptome data, we also screened for genes related to carotenoid synthesis, ZEP, PSY. carotenoids, as natural pigments that are widely available in nature, can present a variety of colors such as orange and yellow (Yuan et al., 2015) can also have a contributing effect on the formation of leaf color traits in yellow leaf, and the large amount of synthesis of ZEP, PSY, promotes the accumulation of carotenoids in yellow leaf, increasing the basal color development of yellow. This is consistent with the fact that in tomato, PSY determines the color of ripe fruits. (Orlovskaya et al., 2018) It is worth mentioning that we identified the cytoskeletal protein filamin gene in our transcriptomic data, which does not directly affect pigmentation per se, but it was found in rice and Arabidopsis studies that FLN may lead to morphological abnormalities in chloroplasts, resulting in a lack of chlorophyll in their structure, and consequently an albino phenotype (Wang et al., 2016). In contrast to green leaf, the high expression of this gene in yellow leaf resulted in reduced chlorophyll content. yellow leaf’s gold leaf trait formation combines multiple factors such as cellular structural conditions, decreased degradation and synthesis of chlorophyll, carotenoid metabolism, and accumulation of flavonoids. These genes drive the transition from green to golden color of leaves through synergistic effects. Meanwhile, this study revealed a series of key candidate genes through transcriptomics, which provided clues for subsequent functional studies.

Although several structural genes differentially expressed in pigment metabolic pathways were identified in this study, the upstream transcriptional mechanisms regulating the expression of these structural genes remain unclear. To further elucidate the transcriptional regulatory network behind the leaf color changes, we screened and analyzed the differentially expressed transcription factors in the yellow-leaved and green-leaved samples.

Among the differentially expressed genes screened, a total of 692 transcription factors were identified, mainly including family members of bHLH, MYB, WRKY, NAC, ERF, and GATA, among which 588 transcription factors showed significant expression changes in yellow- versus green-leaved samples (|log2FoldChange| ≥ 1, padj < 0.01). Notably, bHLH family KI387_035757 and KI387_019382 were highly up-regulated in yellow leaf (log2FoldChange > 12), whereas NAC family members KI387_006865 was significantly down-regulated and KI387_024892 was up-regulated, suggesting that the NAC transcription factors may be important in the regulation of chlorophyll degradation and leaf senescence. Multiple ERF family members also showed significant up- or down-regulated changes, suggesting that they may have complex functions in secondary metabolism regulation and stress response. In addition, GATA family member KI387_005364 was significantly up-regulated in yellow leaf, which may be involved in the process of leaf color formation by affecting light signaling and chlorophyll biosynthesis pathways. It has been shown that bHLH transcription factors directly regulate anthocyanin biosynthesis in Arabidopsis (Nesi et al., 2000); the NAC transcription factor VNI2 affects leaf senescence and chlorophyll metabolism through the regulation of downstream responding genes (Yang et al., 2011); and the ERF transcription factor plays an important regulatory role during abiotic stress and growth and development (Xie et al., 2019); and GATA transcription factors have important functions in photosynthesis and chloroplast development (Hudson et al., 2011).

Combined with the transcriptome data from this study, it is speculated that these transcription factors may synergistically contribute to the gold leaf phenotype by regulating flavonoid metabolism, chlorophyll degradation and light signaling pathways. However, this speculation still needs to be further confirmed by subsequent functional validation experiments.

### 4.2 Metabolomic insights into yellow leaf formation

Metabolomics has the potential to reveal the molecular mechanisms underlying plant phenotypic differences through the direct identification and quantification of multiple metabolites (Li et al., 2020). Although metabolomics has been used to study leaf coloration in a wide range of plants, our study is the first time that metabolomics approaches have been applied to the study of leaf color variation in Northeast Picea abies, and we chose the most classical type of variation, YELLOW leaf, We chose the most classical variation type yellow leaf and compared it with the natural species green leaf to elucidate the metabolomics basis of leaf color variation. We found that the main compounds involved in leaf color formation were tetrapyrroles and flavonols. Among them, quercetin, kaempferol, and Ridiculuflavone D were the main pigments in yellow leaf. Flavonoids are the most abundant secondary metabolites in plants, which not only provide protection against ultraviolet light, pests and diseases, but also play a crucial role in tissue coloration (Dwibedi et al., 2022). Based on structural differences, common flavonols can be categorized as quercetin, kaempferol, populin, and isorhamnetin, etc. F3H plays a key role in flavonol biosynthesis and has been shown to play an important role in yellowing of ginkgo biloba and “HAES344” macadamia nut leaves, affecting pigmentation (Yang et al., 2023, 2024, 2025). Metabolomics data showed that the levels of kaempferol 3-p-coumarate, quercetin-3’-glucuronide, and kaempferol-3-O-rutinoside were significantly higher than those of the control type. The accumulation of these metabolites promoted the expression of yellowing traits in yellow leaf. This result is consistent with the discovery of the major components of Camellia sinensis (Jiang et al., 2024). Meanwhile, the down-regulation of chlorophyll c and up-regulation of Phaeophorbide b in tetrapyrrole compounds promoted the degradation of chlorophyll b, which weakened the green coloration in yellow leaf. In addition, some other tetrapyrroles and their derivatives, such as Pheophorbide A, Epoxypheophorbide a, and 13-epi-Phaeophorbide a methyl ester showed down-regulated expression in yellow leaf. These metabolites are products of direct degradation of chlorophyll a (Das et al., 2018). This result suggests that the expression of gold leaf traits in yellow leaf is associated with the accumulation of flavonoids together with the degradation of chlorophyll B. Meanwhile, the metabolomics data also revealed intermetabolite correlations among individual metabolites, and the results provided important insights into the synergistic or mutually exclusive roles of metabolites, and we found that flavonoids were negatively correlated with tetrapyrroles, including chlorophyll metabolites, suggesting that the accumulation of flavonoids and the degradation of chlorophyll are jointly involved in the regulation of gold leaf traits (Mattila et al., 2018; Wu et al., 2020a). This synergistic effect may confer a distinctive golden color to yellow leaf by altering light absorption and reflection properties. Differential metabolite and chord diagrams further revealed the complex interactions between metabolites. Amino acids showed synergistic regulation with chlorophyll metabolites, which may be related to the role of amino acids in chlorophyll synthesis and degradation (Liu et al., 2023). However, amino acids showed negative correlation with flavonoids, which may reflect the competitive allocation of resources among different secondary metabolic pathways (Wu et al., 2016). Notably, paclitaxel synthesis was closely associated with the expression of other key metabolites. The analysis showed that high paclitaxel expression was significantly positively correlated with the accumulation of flavonoids and the up-regulation of some chlorophyll metabolites, whereas it was negatively correlated with the degradation of amino acids and organic oxides. This suggests that the metabolic network of paclitaxel may share regulatory factors with the regulatory mechanisms of photosynthetic pigments and secondary metabolites. Flavonoids may indirectly contribute to paclitaxel accumulation through their antioxidant and photoprotective functions (Agati et al., 2011). In addition, degradation of chlorophyll may release metabolic intermediates that provide precursors or energy support for secondary metabolism.

### 4.3 Combined metabolomics and transcriptomics analysis

In this study, we screened a total of 9 differential genes with 20 differential metabolites for correlation analysis among different leaf color types of NE Picea abies. Among the key differential genes screened in this study, GLK (Golden-like) and SGR (Stay-green) were directly involved in the regulation of chlorophyll content, and both showed significant synergistic effects with the synthesis and degradation process of tetrapyrrole compounds. This result is consistent with existing studies that GLK, as an important regulator of chloroplast development, positively regulates chloroplast synthesis, whereas SGR promotes chlorophyll degradation (Luo et al., 2023). In addition, the differences in the accumulation levels of tetrapyrrole compounds associated with chlorophyll metabolism in different types suggest that chlorophyll degradation may be one of the important factors contributing to the differences in leaf color.

Flavonoids play an important role in plant secondary metabolism and have a key influence in leaf color formation. In this study, we found that differential expression of the C3H (coumarate 3-hydroxylase) gene family was highly correlated with flavonoi accumulation, and in particular, the expression levels of F3H (flavonoid 3-hydroxylase) and FLS (flavonol synthase) were significantly and positively correlated with most of the flavonoids. This result supports the central role of F3H and FLS in flavonoid biosynthesis (Falcone Ferreyra et al., 2012). In contrast, the DFR (bifunctional anthocyanin reductase) gene showed a positive correlation with tetrapyrroles and a negative correlation with flavonoids in this study, which may indicate that DFR competitively influences the biosynthetic partitioning of flavonoids and anthocyanins (Nakatsuka et al., 2014).

Interestingly, the FLN (Flavone synthase-like protein) gene, which has been reported to cause a yellowing phenotype in rice, also showed a negative correlation with tetrapyrroles and a positive correlation with flavonoids in this study. This phenomenon suggests that FLN may influence different types of leaf color expression in NEH by regulating the balance between tetrapyrrole metabolism and flavonoid accumulation. This result is consistent with previous findings in rice (Qiu et al., 2017), further supporting the potential function of FLN in regulating plant leaf color.

Through transcriptomic, metabolomic, and integrated omics analysis, we conclude that flavonoids play a critical role in the formation of the yellow-leaf phenotype. To further validate the role of flavonoids in leaf color formation, we conducted flavonoid content measurements. The results confirmed that, although the total flavonoid content in the yellow-leaf variant was lower, the relative proportion of flavonoids in the total pigment content was significantly higher than in the green-leaf phenotype. This supports our hypothesis that flavonoid accumulation is a key factor in the transition from green to yellow coloration. The combined results from transcriptomics, metabolomics, and flavonoid content measurement provide a comprehensive understanding of the molecular mechanisms underlying leaf color variation in *Taxus cuspidata*. These findings suggest that flavonoids, in conjunction with carotenoids and chlorophyll, act through complex and coordinated metabolic pathways to drive the formation of the yellow-leaf phenotype.

## 5 Conclusion

In this study, the molecular basis for the formation of gold leaf traits in Picea abies (Picea abies) was systematically revealed through the combined analysis of transcriptome and metabolomics. The results showed that there was a significant accumulation of flavonoid metabolites in the golden leaf type “yellow leaf”, accompanied by an increase in chlorophyll degradation products and a decrease in tetrapyrrole compounds, which showed a typical golden leaf pigment profile. At the gene expression level, key genes involved in flavonoid and carotenoid synthesis, such as F3H, FLS, FLN, ZEP and PSY, were significantly up-regulated, whereas genes related to anthocyanin synthesis, such as GLK and SGR (genes that regulate the synthesis and degradation of chlorophyll) as well as DFR, were down-regulated, suggesting that the formation of gold leaf was a result of the synergistic effect of the upstream enhancement of flavonoids and downstream chlorophyll degradation. qRT-PCR verified the expression trends of core genes. and KEGG pathway enrichment further supported this metabolic regulation pattern. Finally, we conducted pigment content analysis at the physiological level to validate our findings. The results confirmed that flavonoids, as key metabolites, play a major role in regulating the formation of the yellow-leaf phenotype, in conjunction with chlorophylls and carotenoids. This further supports the hypothesis that flavonoid accumulation, together with changes in chlorophyll and carotenoid content, contributes to the yellow leaf trait in *Taxus cuspidata*.

## Data Availability

The sequencing data generated in this study have been deposited in the NCBI Sequence Read Archive (SRA) under the BioProject accession number PRJNA1306783. This BioProject contains eight sequencing runs (SRR35012958–SRR35012965), corresponding to the transcriptome sequencing of *Taxus cuspidata*. The data are publicly accessible at the following link: https://www.ncbi.nlm.nih.gov/bioproject/PRJNA1306783.

## References

[B1] Agati G. Biricolti S. Guidi L. Ferrini F. Fini A. Tattini M. (2011). The biosynthesis of flavonoids is enhanced similarly by UV radiation and root zone salinity in L. vulgare leaves. J. Plant Physiol. 168, 204–212. doi: 10.1016/j.jplph.2010.07.016 20850892

[B2] Chen Y. Han Y. He S. Cheng Q. Tong H. (2024). Differential metabolic profiles of pigment constituents affecting leaf color in different albino tea resources. Food Chem. 467, 142290. doi: 10.1016/j.foodchem.2024.142290 39637662

[B3] Cutolo E. A. Guardini Z. Dall’Osto L. Bassi R. (2023). A paler shade of green: engineering cellular chlorophyll content to enhance photosynthesis in crowded environments. New Phytol. 239, 1567–1583. doi: 10.1111/nph.19064, PMID: 37282663

[B4] Das A. Christ B. Hörtensteiner S. (2018). Characterization of the pheophorbide a oxygenase/phyllobilin pathway of chlorophyll breakdown in grasses. Planta 248, 875–892. doi: 10.1007/s00425-018-2946-2, PMID: 29951845

[B5] Dong N.-Q. Lin H.-X. (2020). Contribution of phenylpropanoid metabolism to plant development and plant-environment interactions. J. Integr. Plant Biol. 63, 180–209. doi: 10.1111/jipb.13054, PMID: 33325112

[B6] Dong H. Xu B. Ji K. (2019). Comparative transcriptome analysis of genes involved in response to thermal stress and leaf colour change of Acer palmatum. Scientia Hortic. 255, 77–85. doi: 10.1016/j.scienta.2019.05.021

[B7] Dwibedi V. Jain S. Singhal D. Mittal A. Rath S. K. Saxena S. (2022). Inhibitory activities of grape bioactive compounds against enzymes linked with human diseases. Appl. Microbiol. Biotechnol. 106, 1399–1417. doi: 10.1007/s00253-022-11801-9, PMID: 35106636

[B8] Falcone Ferreyra M. L. Rius S. P. Casati P. (2012). Flavonoids: biosynthesis, biological functions, and biotechnological applications. Front. Plant Sci. 3. doi: 10.3389/fpls.2012.00222, PMID: 23060891 PMC3460232

[B9] Fan Y.-G. Zhao T.-T. Xiang Q. Han X. Yang S.-S. Zhang L.-X.. (2024). Multi-omics research accelerates the clarification of the formation mechanism and the influence of leaf color variation in tea (Camellia sinensis) plants. Plants 13, 426. doi: 10.3390/plants13030426, PMID: 38337959 PMC10857240

[B10] Fang H. Dong Y. Yue X. Chen X. He N. Hu J.. (2019). MdCOL4 interaction mediates crosstalk between UV-B and high temperature to control fruit coloration in apple. Plant Cell Physiol. 60, 1055–1066. doi: 10.1093/pcp/pcz023, PMID: 30715487

[B11] Gesell A. Blaukopf M. Madilao L. Yuen M. Withers S. Mattsson J.. (2015). The gymnosperm cytochrome P450 CYP750B1 catalyzes stereospecific monoterpene hydroxylation of (+)-sabinene in thujone biosynthesis in western redcedar1. Plant Physiol. 168, 106–194. doi: 10.1104/pp.15.00315, PMID: 25829465 PMC4424034

[B12] Gould K. (2012). Anthocyanins: biosynthesis, functions, and applications. Natural Products (1).

[B13] Hörtensteiner S. (2009). Stay-green regulates chlorophyll and chlorophyll-binding protein degradation during senescence. Trends Plant Sci. 14, 155–162. doi: 10.1016/j.tplants.2009.01.002, PMID: 19237309

[B14] Hudson D. Guevara D. Yaish M. Hannam C. Long N. Clarke J.. (2011). GNC and CGA1 modulate chlorophyll biosynthesis and glutamate synthase (GLU1/Fd-GOGAT) expression in Arabidopsis. PLoS One 6, e26765. doi: 10.1371/journal.pone.0026765, PMID: 22102866 PMC3213100

[B15] Huo J. Zhang N. Gong Y. Bao Y. Li Y. Zhang L.. (2024). Effects of different light intensity on leaf color changes in a Chinese cabbage yellow cotyledon mutant. Front. Plant Sci. 15. doi: 10.3389/fpls.2024.1371451, PMID: 38689838 PMC11058996

[B16] Jiang L. Han L. Zhang W. Gao Y. Xu X. Chen J.. (2024). Elucidation of the key flavonol biosynthetic pathway in golden Camellia and its application in genetic modification of tomato fruit metabolism. Horticulture Res. 12, uhae308. doi: 10.1093/hr/uhae308, PMID: 39944987 PMC11818005

[B17] Li W. Li Q. Che J. Ren J. Wang A. Chen J. (2024). A key R2R3-MYB transcription factor activates anthocyanin biosynthesis and leads to leaf reddening in poplar mutants. Plant Cell Environ. 48, 2067–2082. doi: 10.1111/pce.15276, PMID: 39558461

[B18] Li W. Yang S.-B. Lu Z. He Z.-C. Ye Y.-L. Zhao B.-B.. (2018). Cytological, physiological, and transcriptomic analyses of golden leaf coloration in Ginkgo biloba L. Horticulture Res. 5. doi: 10.1038/s41438-018-0015-4, PMID: 29507736 PMC5830439

[B19] Li B.-J. Zheng B. Wang J.-Y. Tsai W. Lu H.-C. Zou L.. (2020). New insight into the molecular mechanism of colour differentiation among floral segments in orchids. Commun. Biol. 3. doi: 10.1038/s42003-020-0821-8, PMID: 32111943 PMC7048853

[B20] Liu D. Wei K. Zhang C. Liu H. Gong Y. Ye Y.. (2023). The potential effects of chlorophyll-deficient mutation and tree_age on the accumulation of amino acid components in tea plants. Food Chem. 411, 135527. doi: 10.1016/j.foodchem.2023.135527, PMID: 36701915

[B21] Lloyd A. Brockman A. Aguirre L. Campbell A. Bean A. Cantero A.. (2017). Advances in the MYB-bHLH-WD repeat (MBW) pigment regulatory model: addition of a WRKY factor and co-option of an anthocyanin MYB for betalain regulation. Plant Cell Physiol. 58, 1431–1441. doi: 10.1093/pcp/pcx075, PMID: 28575507 PMC5914458

[B22] Lu L. Fritsch P. Matzke N. Wang H. Kron K. Li D. Z.. (2019). Why is fruit colour so variable? Phylogenetic analyses reveal relationships between fruit-colour evolution, biogeography and diversification. Global Ecol. Biogeography 28, 891–903. doi: 10.1111/geb.12900

[B23] Luo J. Abid M. Zhang Y. Cai X. Tu J. Gao P.. (2023). Genome-wide identification of kiwifruit SGR family members and functional characterization of SGR2 protein for chlorophyll degradation. Int. J. Mol. Sci. 24, 1993. doi: 10.3390/ijms24031993, PMID: 36768313 PMC9917040

[B24] Martens S. Preuss A. Matern U. (2010). Multifunctional flavonoid dioxygenases: flavonol and anthocyanin biosynthesis in Arabidopsis thaliana L. Phytochemistry 71, 1040–1049. doi: 10.1016/j.phytochem.2010.04.016, PMID: 20457455

[B25] Mattila H. Valev D. Havurinne V. Khorobrykh S. Virtanen O. Antinluoma M.. (2018). Degradation of chlorophyll and synthesis of flavonols during autumn senescence—the story told by individual leaves. AoB Plants 10, ply028. doi: 10.1093/aobpla/ply028, PMID: 29977486 PMC6007487

[B26] Nakatsuka T. Sasaki N. Nishihara M. (2014). Transcriptional regulators of flavonoid biosynthesis and their application to flower color modification in Japanese gentians. Plant Biotechnol. 31, 389–399. doi: 10.5511/plantbiotechnology.14.0731a

[B27] Nesi N. Debeaujon I. Jond C. Pelletier G. Caboche M. Lepiniec L. (2000). The TT8 gene encodes a basic helix-loop-helix domain protein required for expression of DFR and BAN genes in arabidopsis siliques. Plant Cell 12, 1863–1878. doi: 10.1105/tpc.12.10.1863, PMID: 11041882 PMC149125

[B28] Orlovskaya O. Vakula S. Khotyleva L. Kilchevsky A. (2018). Association between total carotenoid content of maize kernels (Zea mays L.) and polymorphic site INDEL1 in PSY1 gene. Russian J. Genetics: Appl. Res. 8, 74–79. doi: 10.1134/s2079059718010112

[B29] Özbayram A. K. Çiçek E. (2018). Thinning experiments in narrow-leaved ash (Fraxinus angustifolia Vahl.) plantations: 10-year results. New Forests 49, 585–598. doi: 10.1007/s11056-018-9642-8

[B30] Qiu Z. Kang S. He L. Zhao J. Zhang S. Hu J.. (2017). The newly identified heat-stress sensitive albino 1 gene affects chloroplast development in rice. Plant Sci. 267, 168–179. doi: 10.1016/j.plantsci.2017.11.015, PMID: 29362095

[B31] Shen J. Zou Z. Zhang X. Zhou L. Wang Y. Fang W.. (2018). Metabolic analyses reveal different mechanisms of leaf color change in two purple-leaf tea plant (Camellia sinensis L.) cultivars. Horticulture Res. 5, 7. doi: 10.1038/s41438-017-0010-1, PMID: 29423237 PMC5802758

[B32] Shi J.-y. Zou X.-b. Zhao J.-w. Mel H. Wang K.-l. Wang X.. Determination of total flavonoids content in fresh Ginkgo biloba leaf with different colors using near infrared spectroscopy. Spectrochimica Acta Part A: Mol. Biomolecular Spectroscopy. doi: 10.1016/j.saa.2012.03.078, PMID: 22522302

[B33] Wang X. Guo Y. Qi W. Zhen L. Yao Y. Qin F. (2022). Compensatory growth and understory soil stoichiometric features of Hippophae rhamnoides at different stubble heights. PeerJ 10, e13363. doi: 10.7717/peerj.13363, PMID: 35855429 PMC9288824

[B34] Wang M. Jiang L. Da Q. Liu J. Feng D. Wang J.. (2016). DELAYED GREENING 238, a nuclear-encoded chloroplast nucleoid protein, is involved in the regulation of early chloroplast development and plastid gene expression in Arabidopsis thaliana. Plant Cell Physiol. 57, 2586–2599. doi: 10.1093/pcp/pcw172, PMID: 27818379

[B35] Wang P. Nguyen K. C. Lindsey J. (2019). Synthesis of the ring C pyrrole of native chlorophylls and bacteriochlorophylls. J. Organic Chem. 84, 11286–11293. doi: 10.1021/acs.joc.9b01650, PMID: 31432671

[B36] Wang Y. Zhen J. Che X. Zhang K. Zhang G. Yang H.. (2023). Transcriptomic and metabolomic analysis of autumn leaf color change in Fraxinus angustifolia. PeerJ 11, e15319. doi: 10.7717/peerj.15319, PMID: 37197583 PMC10184661

[B37] Wu Y. Guo J. Wang T. Cao F. Wang G. (2020b). Metabolomic and transcriptomic analyses of mutant yellow leaves provide insights into pigment synthesis and metabolism in Ginkgo biloba. BMC Genomics 21. doi: 10.1186/s12864-020-07259-6, PMID: 33267778 PMC7709416

[B38] Wu H. Shi N. An X. Liu C. Fu H. Cao L.. (2018). Candidate Genes for Yellow Leaf Color in Common Wheat (Triticum aestivum L.) and Major Related Metabolic Pathways according to Transcriptome Profiling. Int. J. Mol. Sci. 19, 1594. doi: 10.3390/ijms19061594, PMID: 29843474 PMC6032196

[B39] Wu J. Wang X. Liu Y. Du H. Shu Q. Su S.. (2016). Flavone synthases from Lonicera japonica and L. macranthoides reveal differential flavone accumulation. Sci. Rep. 6, 19245. doi: 10.1038/srep19245, PMID: 26754912 PMC4709722

[B40] Wu M. Xu X. Hu X. Liu Y. Cao H. Chan H.. (2020a). SlMYB72 regulates the metabolism of chlorophylls, carotenoids, and flavonoids in tomato fruit1. Plant Physiol. 183, 854–868. doi: 10.1104/pp.20.00156, PMID: 32414899 PMC7333684

[B41] Xie Z. Nolan T. M. Jiang H. Yin Y. (2019). AP2/ERF transcription factor regulatory networks in hormone and abiotic stress responses in Arabidopsis. Front. Plant Sci. 10. doi: 10.3389/fpls.2019.00228, PMID: 30873200 PMC6403161

[B42] Yang W. Li X. Xiao Q.-s. Hong X. Shao Q. Chen N. (2024). Exploration of mechanism underlying the lipid alterations in the yellowing leaves of ‘HAES344’ macadamia. Scientia Hortic. 336, 113435. doi: 10.1016/j.scienta.2024.113435

[B43] Yang W. Ouyang Q. Chen J. Zeng L. Hong X. Li X.. (2025). Integrated transcriptomic and metabolomic insights into ascorbate biosynthesis and glutathione metabolism during leaf yellowing in ‘HAES344’ macadamia. Scientia Hortic. 342, 114051. doi: 10.1016/j.scienta.2025.114051

[B44] Yang S.-D. Seo P. Yoon H.-K. Park C.-M. (2011). The Arabidopsis NAC Transcription Factor VNI2 Integrates Abscisic Acid Signals into Leaf Senescence via the COR/RD Genes. Plant Cell 23, 2155–2168. doi: 10.1105/tpc.111.084913, PMID: 21673078 PMC3160032

[B45] Yang W. Xu H. Xiao Q. Li X. Shao Q. (2023). Combined analysis of metabolome and transcriptome provides insights into metabolisms of chlorophylls, carotenoids, and flavonoids in the yellowing leaves of ‘HAES344’ macadamia. Scientia Hortic. 308, 111600. doi: 10.1016/j.scienta.2022.111600

[B46] Yuan H. Zhang J. Nageswaran D. C. Li L. (2015). Carotenoid metabolism and regulation in horticultural crops. Horticulture Res. 2. doi: 10.1038/hortres.2015.36, PMID: 26504578 PMC4591682

[B47] Zhang y. (2021). Taxus cuspidata: Variation and Breeding of Cultivars (Shenyang: Liaoning Science and Technology Press).

[B48] Zhao M. Li X. Zhang X. Zhang H. Zhao X. (2020). Mutation mechanism of leaf color in plants: A review. Forests 11, 851. doi: 10.3390/f11080851

[B49] Zhao D. Tao J. (2015). Recent advances on the development and regulation of flower color in ornamental plants. Front. Plant Sci. 6. doi: 10.3389/fpls.2015.00261, PMID: 25964787 PMC4410614

[B50] Zhao D. Tao J. Han C. Ge J. (2012). Flower color diversity revealed by differential expression of flavonoid biosynthetic genes and flavonoid accumulation in herbaceous peony (Paeonia lactiflora Pall.). Mol. Biol. Rep. 39, 11263–11275. doi: 10.1007/s11033-012-2036-7, PMID: 23054003

